# Promising Results With NAD Supplementation in Rare Diseases With Premature Aging and DNA Damage

**DOI:** 10.1111/acel.70319

**Published:** 2025-12-23

**Authors:** Vilhelm A. Bohr

**Affiliations:** ^1^ Department of ICMM University of Copenhagen Copenhagen Denmark

## Abstract

Nicotinamide adenine dinucleotide (NAD) has garnered significant attention in recent years due to its central role in cellular metabolism and its potential as a supplement to promote health and longevity. While numerous human studies indicate that NAD supplementation offers benefits with minimal or no side effects, some studies show no observable advantages. This discrepancy highlights the importance of identifying individuals who are most likely to benefit from NAD‐based interventions. One critical factor in the efficacy of NAD supplementation relates to its declining levels in certain individuals, driven by various causes of NAD depletion. NAD is a vital substrate for numerous enzymatic processes, notably those involving poly‐ADP‐ribose polymerase (PARP) enzymes. PARP enzymes, especially PARP1, play a pivotal role in DNA repair by detecting and signaling DNA damage. Excessive activation of PARP, hyperparylation, is frequently observed in DNA repair disorders where DNA damage accumulates due to defective repair mechanisms. This hyperparylation has been implicated in the pathogenesis of several premature aging diseases. Such conditions often involve defective DNA repair pathways, elevated parylation levels, and associated mitochondrial dysfunction, factors that contribute to accelerated cellular aging. In model systems that mimic these disorders, as well as in emerging human studies, NAD supplementation has demonstrated promising benefits, including improved DNA repair capacity and improved mitochondrial function. These findings suggest that NAD supplementation could serve as an effective intervention for rare genetic diseases characterized by premature aging and DNA repair deficiencies. More broadly, these insights open new avenues for general aging research.

## Graphical abstract

Increased DNA damage leads to increased parylation, causing mitochondrial dysfunction. This happens when DNA repair is defective, for example in known diseases and DNA repair capacity varies between individuals, some being at higher risk for hyperparylation.

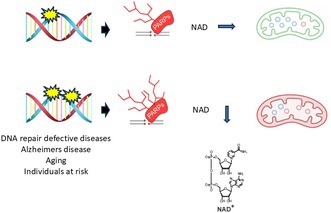

## 1 DNA damage, NAD Levels and Aging

**FIGURE 1:**
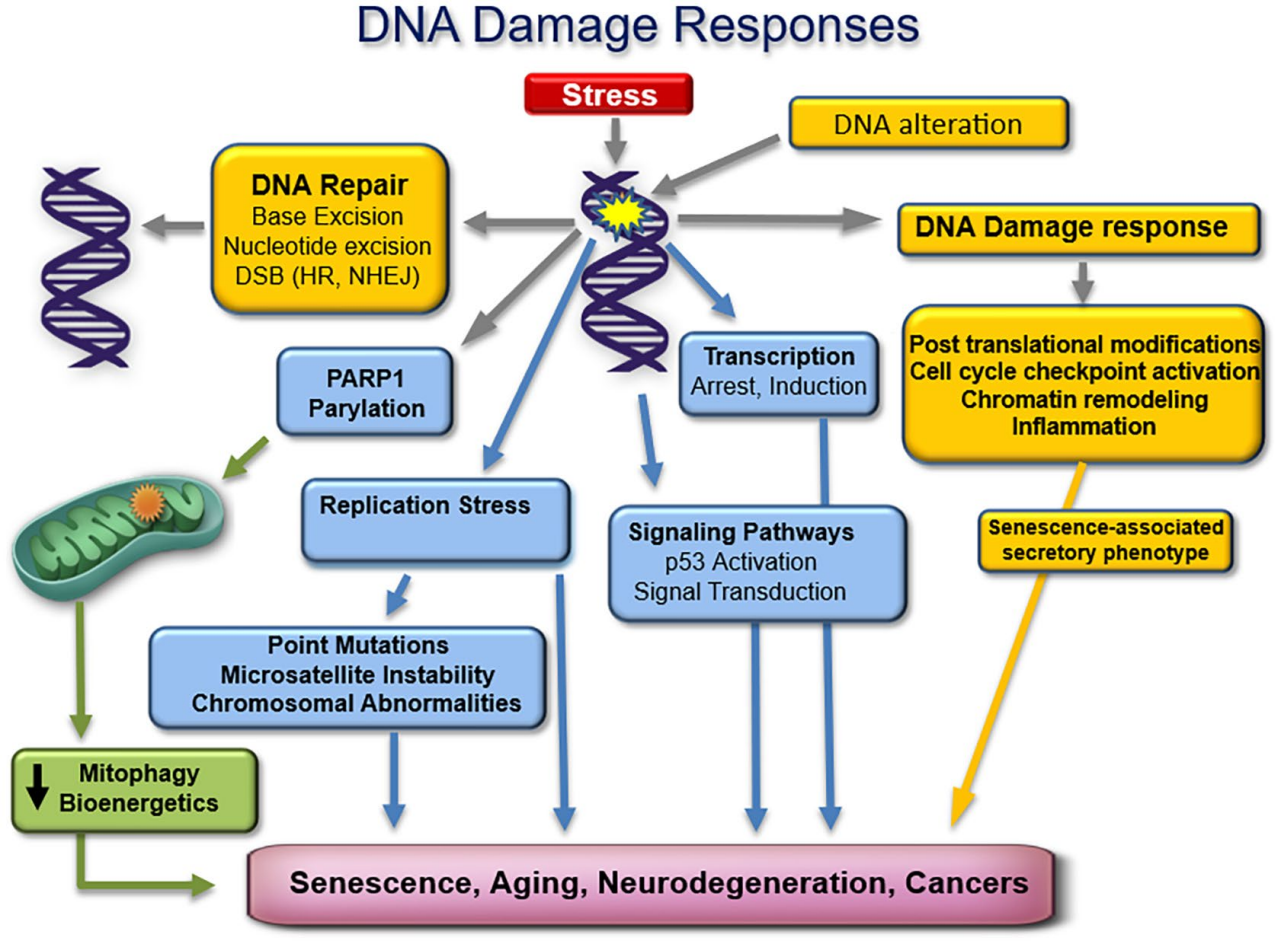
Illustrates some key pathways in the DNA damage response. DNA damage affects a large number of pathways and has profound effects. There is direct signaling from lesions in the DNA to DNA repair and replication processes, PARP parylation signaling, and then signaling processes through mitochondria, all underlying the response signaling system involved in cancer, neurodegeneration, and aging.

DNA damage of many kinds constantly accumulate in the genome at high frequencies and has many cellular consequences as shown in Figure 1.

The cellular implications of NAD functions are profound. NAD levels fluctuate in response to physiological and pathological conditions, significantly impacting cellular metabolism, stress responses, and survival mechanisms (Verdin 2015; Amjad et al. 2021). This is particularly evident in tissues with high energy demands such as the brain and skeletal muscle. Extensive research has demonstrated that NAD levels decline with age across multiple species, correlating with various hallmarks of aging, including mitochondrial dysfunction, genomic instability, and altered cellular communication. This age‐related decline in NAD+ has been observed in rodents (Yoshino et al. 2018) and nematodes (Mouchiroud et al. 2013), suggesting a conserved mechanism potentially contributing to age‐associated functional decline (McReynolds et al. 2020; Migaud et al. 2024). In human tissues, the decline of NAD with aging is not uniform and still needs further investigation (reviewed in Vinten et al. (2025)). Studies have shown a decrease with aging in human skin (Massudi et al. 2012), liver (Zhou et al. 2016) and brain (Zhu et al. 2015), but other tissues show no decline.

There have been several clinical studies using NAD supplementation, mostly nicotinamide riboside (NR, Niagen), but also NMN (Damgaard and Treebak 2023; Migaud et al. 2024). An updated list of published clinical trials was recently reported in a review by Vinten et al. (2025). A number of studies using NAD supplementation have resulted in improved clinical features and increased NAD levels in blood or tissues, and other studies have shown no measurable benefits. Among neurodegenerative diseases, there are promising results both at the preclinical and clinical levels. This includes many positive results in mouse models with Alzheimer's disease (AD) (Hou et al. 2018) (Wang et al. 2022) and in Parkinson's disease patients (Dölle and Tzoulis 2025). Other clinical studies have shown no improvement (Damgaard and Treebak 2023; Migaud et al. 2024). Clinical areas, including neurodegeneration, vision, hearing, and inflammation, appear to benefit from NAD supplementation, whereas musculoskeletal conditions appear to be less receptive to this intervention (Vinten et al. 2025; Zhang, Wang, et al. 2025). The safety record of NAD supplementation in model systems and humans appears to be very high, with none or very limited side effects reported (Migaud et al. 2024). Doses in NR clinical studies have generally been about 1 g per day, but up to 3 g per day appear to be safe (Berven et al. 2023).

## 2 Cellular NAD Pools

The differential intracellular distribution of enzymes contributing to NAD replenishment or degradation suggests that each subcellular organelle possesses a specific mechanism for maintaining NAD levels. This notion is supported by the finding that segregated NAD pools have been demonstrated in mitochondria, nuclei, peroxisomes, the Golgi apparatus, and the endoplasmic reticulum (VanLinden et al. 2015; Høyland et al. 2024). Such compartmentalization of metabolites and small molecules within the cell is not uncommon as calcium ions, ATP (Wright et al. 2016), and acetyl‐CoA (Bulusu et al. 2017) are also compartmentalized. The NAD exchange mechanisms between the cytosol and nucleus are poorly understood. However, the nuclear pore would not be expected to impose a physical barrier to NAD diffusion between the two compartments (Knockenhauer and Schwartz 2016), as the selective depletion of NMNAT2 or NMNAT1 limits NAD availability only in the cytosol or only in the nucleus (Ryu et al. 2018).

Higher activity of poly‐ADP‐ribosyl polymerase 1 (PARP‐1) and more PARylated proteins have been detected in the mitochondrial fraction than in the nucleus. Mitochondria possess their own NAD^+^ pool, implying the possibility of utilizing NAD+ from the mitochondria for PARP‐1 functions (Kadam et al. 2020; Lee et al. 2022). Missing mitochondrial membrane potential yielded by PARP‐1 overexpression suggests that PARP‐1 maintains mitochondrial function under physiological conditions (Jubin et al. 2016). PARP1 was reported to be localized only in the nucleus. However, reports are now suggesting the presence of intramitochondrial PARP1 (mtPARP1) (Rossi et al. 2009; Szczesny et al. 2014; Lee et al. 2022). Its presence may depend on cellular circumstances. Potential roles of PARP‐1 in mitochondrial processes include participation in bioenergetics, mitochondrial DNA (mtDNA) repair, cell fate, mitophagy (Vida et al. 2016), and transcription, as it associates with the major transcription factor TFAM (Lee et al. 2022). It has been suggested that PARP‐1 is involved in multiple cellular events related to PAR‐dependent mitochondrial lesions. Hyperactivation of PARP‐1 in response to extensive DNA damage and/or oxidative stress decreases cytosolic NAD^+^ levels, which impedes glycolysis and damages the electron transport chain (Strømland et al. 2019). The altered glucose flux suppresses the delivery of pyruvate and nicotinamide adenine dinucleotide+ hydrogen (NADH) to the mitochondria, thereby contributing to mitochondrial depolarization by starving mitochondria of energy substrate (Strømland et al. 2019). The mtPARP1 Km value for NAD (22 μm) is significantly lower than the Km value of nuclear PARP1 (210 μm) (Burzio et al. 1981; Amé et al. 1999). The mitochondrial NAD levels are higher than the cytosolic levels, with the relative difference being cell‐type dependent (Sauve 2008). In neurons, mitochondrial NAD pools are larger, making up about 50% of the total cellular NAD when compared to astrocytes, where they represent about 25% (Alano et al. 2007; Waddell et al. 2023). A NAD‐specific transporter is contributing to the replenishment of intramitochondrial NAD pools (Girardi et al. 2020; Luongo et al. 2020).

## 3 Diseases of Premature Aging and DNA Repair Deficiencies

Some rare diseases are referred to as premature aging or segmental progerias because their clinical features mirror those of normal aging, but manifest much earlier in life. These disorders are monogenic, resulting from mutations in specific genes, all of which play critical roles in DNA metabolism and DNA repair.

This group includes Werner syndrome, Cockayne syndrome, Xeroderma pigmentosum, Ataxia‐telangiectasia, Bloom syndrome, Rothmund‐Thomson syndrome, and Hutchinson‐Gilford progeria.

*Werner syndrome* is perhaps the most recognized premature aging syndrome. The patients develop aging‐related features in their teenage years and die in their 4th or 5th decade. Over these years they develop many features seen in the normal aging process. This includes graying of the hair, normal aging at an early stage of their life; gray hair, alopecia, muscle atrophy, osteoporosis, cataracts, hypogonadism, diabetes mellitus, hyperlipidemia, atherosclerosis, malignancy, brain atrophy, and senile dementia (Goto 2000). Gene expression profiling of Werner syndrome cells closely resembles that of normal aging (Kyng et al. 2003).

Werner protein (WRN) plays essential roles in DNA metabolism (Croteau et al. 2014; Shamanna et al. 2017; Lu and Davis 2021; Paccosi et al. 2025). It is involved in a number of DNA repair pathways, including DNA double‐strand break repair and base excision repair (Paccosi et al. 2025) and it is a pathway choice protein in some of these pathways (Shamanna et al. 2016). WRN participates in DNA replication (Paccosi et al. 2025), transcription and in telomere maintenance (Opresko et al. 2005). While in vitro and cellular studies clearly show that WRN is a central player in DNA metabolism and therefore of great relevance to the aging process, the mouse models that were developed had none or very few disease features. One of those features was related to telomere function, which aligned with many studies showing that WRN played a role at the telomere (Opresko et al. 2005). However, the overall, broader picture was that mice were not significantly affected or aged differently when they were depleted of WRN. This hampered the interest in WS as a model for aging. Other animal models of WRN, including Drosophila and *C. elegans*, showed more significant aging‐associated defects, but were of less penetrance than the mouse model. In recent years, there have been many studies emphasizing the importance of WRN in DNA metabolism, cancer, and aging (Peng et al. 2025; Zhang, Zhang, et al. 2025).

*Bloom syndrome* is caused by mutations in the BLM gene, a member of the RecQ helicase family as are WRN and ReCQL4. Bloom syndrome (BS), a rare genetic disorder, with < 300 cases reported worldwide is an autosomal hereditary recessive disease characterized by genetic instability and cancer predisposition. Patients with Bloom's syndrome are 150 to 300 times more likely to develop cancers than are normal individuals (Ababou 2021). The wide spectrum of cancers developed by BS patients suggests that early initial events occur in BS cells, which may also be involved in the initiation of carcinogenesis in the general population, and these may be common to several cancers. BLM protein participates in various genome metabolomic functions, including genome surveillance and maintenance through its major roles during DNA replication, DNA repair, and transcription (Ababou 2021).

*Cockayne syndrome* is a rare and fatal autosomal recessive neurodegenerative disorder characterized by growth failure, impaired development of the nervous system, abnormal sensitivity to sunlight, eye disorders, and premature aging (Pearce 1972; Laugel 1993; Tiwari et al. 2021; Afonso‐Reis et al. 2025). There are two complementation groups, A and B. Cockayne syndrome (CS) genes play important roles in DNA repair and transcription. Mutation in these genes results in CS, a profoundly debilitating disorder characterized by short stature, microcephaly, premature aging, neurodegeneration, photosensitivity, vision impairment, hearing loss, and bone and kidney abnormalities (Scheibye‐Knudsen et al. 2012; Karikkineth et al. 2017; Rajamani et al. 2024). While CSA and CBS proteins have different functions, the clinical features of the patients are similar for the two complementation groups (Karikkineth et al. 2017). The molecular basis of CS has traditionally been attributed to defects in transcription and transcription‐coupled nucleotide excision repair (TC‐NER). However, recent work suggests that defects in base excision DNA repair and mitochondrial functions also play important roles (Tiwari et al. 2021). One of the key molecular characteristics of CS (patient‐derived) cells is the reduced abundance of nicotinamide dinucleotide (NAD+) (Scheibye‐Knudsen, Mitchell, et al. 2014). Patients with Cockayne syndrome often suffer from severe neurodegeneration (Ropert et al. 2024), kidney function problems (Pekhale et al. 2025) and in CS model systems, mitochondrial dysfunction is prevalent (Okur et al. 2020; Karikkineth et al. 2017).

*Rothmund‐Thomson Syndrome (RTS)* is a rare, inherited disorder characterized by a distinctive rash, poikiloderma, which appears on the cheeks, arms, and legs in infancy (Martins et al. 2023). RTS also involves a variety of other symptoms, including sparse hair, slow growth, skeletal abnormalities, cataracts, and an increased risk of cancer. The syndrome is most often caused by mutations in the RECQL4 helicase, a member of the RecQ helicase family, which is involved in DNA repair and replication (Croteau et al. 2014). It is one of the early responders in the DNA double‐strand break repair process (Hussain et al. 2025).

*Ataxia‐telangiectasia* (A‐T) is a rare genome instability and neurocutaneous syndrome caused by biallelic mutations in the ataxia‐telangiectasia mutated (ATM) gene, exhibiting an incidence of ~1:40,000–1:300,000 live births in different communities worldwide and a median survival rate of ~25 years (Collyer and Rajan 2024). A‐T is named for its characteristic cerebellar ataxia in the early toddler years and variable oculocutaneous telangiectasias in the school‐age years. While its name hints at neurologic and cutaneous manifestations, this multisystemic disorder also has important immunologic, oncologic, respiratory, and endocrinologic implications (Collyer and Rajan 2024). The ATM protein kinase is primarily known for orchestrating the cellular response to DNA double‐strand breaks (DSBs) (Aguado et al. 2022). Although ATM's nuclear functions related to the maintenance of genome stability remain the best understood aspect of ATM, it has many other roles within the cell (Aguado et al. 2022). The classical clinical picture of A‐T resembles many aspects of normal human aging (Aguado et al. 2022). A‐T is first presented by progressive cerebellar cortical degeneration that begins with deterioration and subsequent loss of Purkinje cells and ultimately affects other cell types leading to the degeneration of the entire cerebellum. The cerebellar ataxia that reflects this process advances into a general motor dysfunction, ultimately limiting most A‐T children to a wheelchair at the late stages of their first decade (Aguado et al. 2022). Immunodeficiency that includes the B‐ and T‐cell lineages is another hallmark and is often accompanied by recurrent pulmonary infections. There is also thymic degeneration, primary gonadal failure, and occasional endocrine abnormalities (Aguado et al. 2022). A‐T plasma analytes and a wide range of clinical abnormalities indicate a strong premature aging component associated with A‐T pathology (Shiloh and Lederman 2017).

*Xeroderma Pigmentosum* is a rare disorder (Rizza et al. 2021) with a strong preponderance for skin and internal cancers. There are several complementation groups, and studies of these individuals led to the discoveries underlying the understanding of UV repair and nucleotide excision repair (NER). Among the several complementation groups of xeroderma pigmentosum, group A, XPA, individuals are particularly prone to severe neurodegeneration, in contrast to individuals with XPC, who do not appear to experience neurodegeneration (Garcia‐Moreno et al. 2023).

## 4 Premature Aging and Neurodegeneration Are Connected to Mitochondrial Deficiency

When analyzing available clinical data from features of the premature aging disorders, we detected mitochondrial involvement in a number of diseases not previously recorded as mitochondrial (Scheibye‐Knudsen et al. 2013). As proof of principle, Cockayne syndrome, ataxia with oculomotor apraxia 1 (AOA1), spinocerebellar ataxia with axonal neuropathy 1 (SCAN1), and ataxia‐telangiectasia were shown to have mitochondrial dysfunction, and those diseases showed a strong alignment with mitochondrial disorders (Scheibye‐Knudsen et al. 2013). We evaluated mitochondrial involvement in aging and detected that accelerated aging disorders with neurodegeneration were associated with mitochondrial dysfunction (Scheibye‐Knudsen et al. 2013). Normal aging was more strongly associated with the mitochondrial diseases than the non‐mitochondrial ones, partially supporting a mitochondrial theory of aging (Scheibye‐Knudsen et al. 2013). This work led to further pursuit of the connection between accelerated aging and mitochondrial dysfunction, and the notion that accelerated aging and DNA repair defects lead to NAD deficits and mitochondrial dysfunction through hyperparylation.

## 5 Hyperparylation in Premature Aging Diseases

Hyperparylation, a condition often associated with DNA repair disorders, where DNA damage accumulates, has been implicated in the pathogenesis of several premature aging diseases. This process involves the excessive addition of poly (ADP‐ribose) chains to proteins, a reaction catalyzed by poly (ADP‐ribose) polymerases (PARPs), mainly PARP1 (Kołacz and Robaszkiewicz 2024). Hyperparylation involves increased PARP1 activity, using NAD as a substrate, and this affects many normal cellular functions, including DNA repair, and leads to further genomic instability (Murata et al. 2019). Extreme hyperparylation can trigger mitochondrial catastrophe, oxidative stress, and ultimately cell death through a non‐canonical form of Parthanatos, which has been described in many neurological diseases (Xu et al. 2023) (Yang et al. 2024).

PARylation can lead to significant depletion of NAD^+^, especially when excessive DNA damage activates PARP1, consuming large amounts of NAD^+^ in the process (Murata et al. 2019). PARylation may cause depletion of NAD pools in the nucleus and in mitochondria since PARP1 is likely also present in mitochondria. This depletion can impact cellular metabolism and energy balance, potentially leading to cell death if NAD^+^ levels become too low (Murata et al. 2019), (Kang et al. 2022). This also leads to various forms of mitochondrial dysfunction and to various forms of excessive stress causing mild to severe cellular dysfunction and even cell death in the form of Parthanatos (Yang et al. 2024).

Excessive parylation leads to DNA damage and deficiencies in DNA repair (Kang et al. 2022). Individuals with DNA repair defects accumulate more DNA damage, resulting in heightened DNA damage responses, including hyperparylation. DNA repair varies widely among individuals, and some conditions, such as certain monogenic disorders, are linked to lower DNA repair levels and premature aging symptoms. In conditions including Werner syndrome and Cockayne syndrome, hyperparylation has been suggested to contribute to the accelerated aging phenotypes observed in patients (Fang, Scheibye‐Knudsen, et al. 2016).

Xeroderma pigmentosum complementation group A (Fang et al. 2014), Cockayne syndrome groups A and B (Scheibye‐Knudsen, Fang, et al. 2014), ataxia telangiectasia model systems (Fang, Kassahun, et al. 2016; Fang and Bohr 2017) and Werner syndrome (Fang et al. 2019) model systems all had significantly lower levels of NAD, presumably because of the increased DNA damage and hyperparylation in these conditions. The model systems included *C. elegans*, human cells, and mouse models. The human cells included mutated diseased cells or knockdowns of the various genes. XPA, Cockayne syndrome, and ataxia telangiectasia patients all have severe neurodegeneration. Werner syndrome patients are not usually known to suffer from neurodegeneration, although schizophrenia has been reported. Also, polymorphisms in the WRN gene have been associated with neurodegeneration (Lebel and Monnat Jr. 2018). Possibly, Werner syndrome patients do not live long enough to develop neurodegeneration.

All of these four diseases of premature aging are associated with mitochondrial dysfunction in the form of defective mitophagy (Fang, Scheibye‐Knudsen, et al. 2016; Croteau et al. 2017; Fang and Bohr 2017; Kerr et al. 2017). Mitophagy is the process of mitochondrial degradation targeting damaged mitochondria. It is an essential process as the accumulation of damaged mitochondria causes severe damage or cell death (Antico et al. 2025; Clague and Urbé 2025; Yang et al. 2025).

**FIGURE 2:**
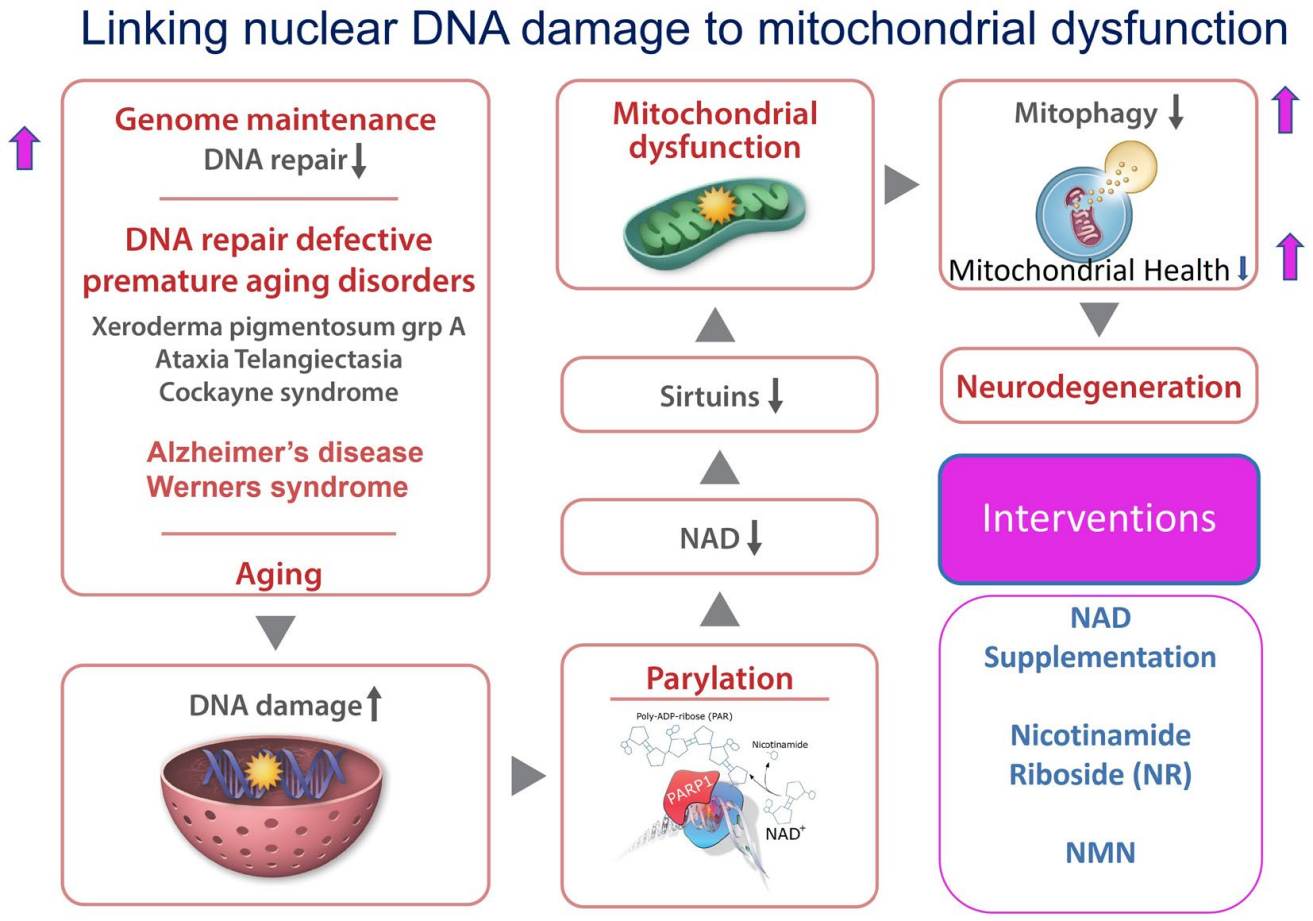
Illustrates how hyperparylation leads to NAD depletion and mitochondrial dysfunction in some rare diseases and in Alzheimer's disease (AD) and possibly in aging in general. Nuclear DNA damage accumulates and elicits hyperparylation, which depletes NAD from various pools in the cell. This leads to loss of function of the other NAD dependent enzymes described above and to mitochondrial dysfunction. The mitochondrial dysfunction commonly detected under these conditions is mitophagy, the process of restoring defective mitochondria via fusion with lysozymes, an essential process in replacing defective mitochondria. Mitophagy has been detected in some of the major neurodegenerative disorders including AD and Parkinson's disease (Hou et al. 2019). After stress and with aging, DNA damage accumulates in the nucleus but also in the mitochondrial DNA. The DNA damage signaling involved in the rare diseases discussed probably comes mainly from the nuclear DNA damage because some of the disease proteins are present in mitochondria while others are not. Thus, this signaling does not involve the disease protein signaling in the mitochondria. For example, Cockayne proteins A and B have been detected in mitochondria (Aamann et al. 2010; Kamenisch et al. 2010) while WRN has not. Intervention with NAD supplementation improves DNA repair and mitophagy. Interventions shown in pink and with pink arrows.

Figure 2 illustrates how NAD decline after DNA damage can lead to mitochondrial dysfunction and how NAD supplementation can be an effective intervention.

### 5.1 Werner Syndrome (WS)

Metabolic dysfunction is a primary feature of WS. We reported impaired mitophagy and depletion of NAD^+^ in WS patient samples and WS invertebrate models (Fang et al. 2019). WRN regulated transcription of a key NAD^+^ biosynthetic enzyme, nicotinamide nucleotide adenylyltransferase 1 (NMNAT1). NAD^+^ repletion restored NAD^+^ metabolic profiles and improved mitochondrial quality through DCT‐1 and ULK‐1‐dependent mitophagy. At the organismal level, NAD^+^ repletion remarkably extended lifespan and delayed accelerated aging, including stem cell dysfunction, in *C. elegans* and *Drosophila melanogaster* models of WS. This indicated that accelerated aging in WS is mediated by impaired mitochondrial function and mitophagy, and that bolstering cellular NAD^+^ levels counteracts WS phenotypes (Fang et al. 2019). In recent work from the Fang lab (Lautrup et al. 2025) loss of WRN increased senescence in mesenchymal stem cells likely related to dysregulation of metabolic and aging pathways. In line with this, NAD^+^ augmentation, via supplementation with nicotinamide riboside, reduced senescence and improved mitochondrial metabolic profiles in mesenchymal stem cells with *WRN* knockout (*WRN* ^*−/−*^) and in primary fibroblasts derived from WS patients compared to controls. Moreover, *WRN* deficiency resulted in decreased mitochondrial NAD^+^ and altered expression of key salvage pathway enzymes, including NMNAT1 and NAMPT (Lautrup et al. 2025).

### 5.2 Cockayne Syndrome

Cockayne syndrome (CS) is an accelerated aging disorder characterized by progressive neurodegeneration. Csb mice were given a high‐fat, caloric‐restricted, or resveratrol‐supplemented diet (Scheibye‐Knudsen, Mitchell, et al. 2014). The high‐fat diet rescued the phenotype of Csb mice at the metabolic, transcriptomic, and behavioral levels. Additional analysis suggests that the premature aging seen in CS mice, nematodes, and human cells results from aberrant PARP activation due to deficient DNA repair leading to decreased SIRT1 activity and mitochondrial dysfunction. β‐hydroxybutyrate levels increased with the high‐fat diet, and β‐hydroxybutyrate, PARP inhibition, or NAD+ supplementation all activated SIRT1 and rescued CS‐associated phenotypes (Scheibye‐Knudsen, Mitchell, et al. 2014). In vitro, CSB displaced activated PARP1 from damaged DNA to limit its activity (Scheibye‐Knudsen, Mitchell, et al. 2014).

PARPs transfer ADP‐ribose from NAD+ to itself, histones, and other proteins at sites of DNA damage. CSA is recruited to stalled RNA pol II by CSB following UV DNA damage (van der Weegen et al. 2020) and is involved in the recruitment of the TC‐NER machinery in response to UV damage via the DDB1‐CUL4‐ E3 ubiquitin ligase complex (Groisman et al. 2003; Fischer et al. 2011). CSB protein displaces PARP1 from its binding sites and terminates its PARylating activity (Fang et al. 2014). Thus, CSB deficiency leads to further depletion of intracellular NAD+ and lowers the activity of sirtuin proteins and other NAD+‐dependent enzymes (Fang, Scheibye‐Knudsen, et al. 2016). This has been observed in cells derived from CS patients and in model animal systems such as mice and worms (Okur et al. 2020).

### 5.3 Ataxia Telangiectasia

Ataxia telangiectasia (A‐T) is a rare autosomal recessive disease characterized by progressive neurodegeneration and cerebellar ataxia. A‐T is causally linked to defects in ATM, a master regulator of the response to and repair of DNA double‐strand breaks. The molecular basis of cerebellar atrophy and neurodegeneration in A‐T patients is unclear. We examined the significance of increased PARylation, low NAD+, and mitochondrial dysfunction in ATM deficient mice and worms (Fang, Kassahun, et al. 2016). Replenishing intracellular NAD+ reduced the severity of A‐T neuropathology, normalized neuromuscular function, delayed memory loss, and extended lifespan in both animal models (Fang, Kassahun, et al. 2016). Treatments that increased intracellular NAD+ also stimulated neuronal DNA repair and improved mitochondrial quality via mitophagy (Fang, Kassahun, et al. 2016). This work linked two major theories on aging, DNA damage accumulation and mitochondrial dysfunction through nuclear DNA damage‐induced nuclear–mitochondrial signaling, and demonstrated that they are important pathophysiological determinants in premature aging of A‐T (Fang, Kassahun, et al. 2016).

Senescence phenotypes and mitochondrial dysfunction are implicated in aging and in premature aging diseases, including ataxia telangiectasia (A‐T). Loss of mitochondrial function can drive age‐related decline in the brain. We showed that mitochondrial dysfunction and cellular senescence with a senescence‐associated secretory phenotype (SASP) occur in A‐T patient fibroblasts, and in ATM‐deficient cells and mice (Yang et al. 2021). Senescence is mediated by stimulator of interferon genes (STING) and involves ectopic cytoplasmic DNA. Boosting intracellular NAD+ levels with nicotinamide riboside (NR) prevented senescence and SASP by promoting mitophagy in a PINK1‐dependent manner. NR treatment also prevented neurodegeneration, suppressed senescence and neuroinflammation, and improved motor function in Atm−/− mice (Yang et al. 2021). This suggested a central role for mitochondrial dysfunction‐induced senescence in A‐T pathogenesis, and that enhancing mitophagy was a potential therapeutic intervention (Yang et al. 2021).

### 5.4 Xeroderma Pigmentosum Group A (XPA)

Mitochondrial dysfunction is a common feature in neurodegeneration and aging. We identified mitochondrial dysfunction in xeroderma pigmentosum group A (XPA), a nucleotide excision DNA repair disorder with severe neurodegeneration, in silico and in vivo (Scheibye‐Knudsen et al. 2013; Fang et al. 2014). XPA‐deficient cells showed defective mitophagy with excessive cleavage of PINK1 and increased mitochondrial membrane potential. The mitochondrial abnormalities appeared to be caused by decreased activation of the NAD+–SIRT1–PGC‐1α axis triggered by hyperactivation of the DNA damage sensor PARP1. This phenotype was rescued by PARP1 inhibition or by supplementation with NAD+ precursors that also rescue the lifespan defect in xpa‐1 nematodes (Fang et al. 2014). Importantly, this pathogenesis appears common to ataxia‐telangiectasia and Cockayne syndrome, two other DNA repair disorders with neurodegeneration, but is absent in XPC, a DNA repair disorder without neurodegeneration. This suggested the presence of a nuclear‐mitochondrial cross‐talk that is critical for the maintenance of mitochondrial health (Fang et al. 2014).

## 6 Results From Clinical Studies with NAD supplementation in Some Rare Disorders

### 6.1 Nicotinamide Riboside Supplementation Benefits in Patients with Werner Syndrome (WS): A Double‐Blind Randomized Crossover Placebo‐Controlled Trial (Shoji et al. 2025)

This 52‐week randomized, double‐blind, placebo‐controlled crossover trial evaluated the safety and efficacy of oral Nicotinamide riboside (NR) supplementation in individuals with Werner syndrome. The study randomized 9 individuals (mean age: 47) who received 1000 mg/day of NR or placebo for 26 weeks before crossing over to the alternate treatment for an additional 26 weeks.

They observed a robust increase in NAD+ levels: NR supplementation led to a ~140% increase in plasma NAD+ levels, compared to a ~4% decrease in the placebo group. Importantly, arterial stiffness was improved in WS patients as NR significantly improved the cardio‐ankle vascular index (CAVI), a measure of arterial stiffness. They observed a cardioprotective lipid shift in WS patients. NR increased the number of large HDL particles, indicating potential cardiovascular benefits. There were signs of wound healing support in WS patients as NR reduced skin ulcer size and heel pad thinning, while ulcers worsened in the placebo group. NR appeared to improve kidney function in WS patients as they had lower urine creatinine. In the study, no moderate or severe adverse events were reported. Mild adverse events were fewer during the NR phase (7) compared to placebo (12). Although mild liver enzyme elevations were noted, they were deemed manageable and consistent with underlying liver sensitivities common in Werner syndrome. This study represents the first clinical evaluation of NR in Werner syndrome and supports further investigation of NAD+ augmentation as a therapeutic strategy in rare progeroid diseases.

### 6.2 Nicotinamide Riboside Improves Ataxia Scores and Immunoglobulin Levels in Ataxia Telangiectasia (Veenhuis et al. 2021)

Treatment of animal models with ataxia telangiectasia (A‐T) with NR improved their neurological outcome and survival. The objective of this study was to investigate the effects of NR in patients with A‐T. This was a single‐center, interventional, open‐label, proof‐of‐concept study where 24 patients with A‐T were treated with NR (25 mg/kg bodyweight per day) during four consecutive months and subsequently followed during 2 months without treatment. During the 6‐month study period, clinical and laboratory parameters were measured. To assess the differences in the individual outcome measures between various time points, a linear mixed model analysis was applied. The effects of NR on ataxia, dysarthria, quality of life, and laboratory parameters were analyzed.

The results of the study were that during treatment, the ataxia scores improved. The mean total Scale for the Assessment and Rating of Ataxia and International Cooperative Ataxia Rating Scale scores decreased to 2.4 and 10.1 points, respectively. Improved ataxia scores (SARA, ICARS) effects reversed after withdrawal. Increased IgG levels were detected in immunodeficient individuals. In immunodeficient patients, the mean serum IgG concentration increased substantially until the end of the study period. Untargeted metabolomics analysis revealed increased plasma levels of NR metabolites and purine nucleosides during treatment. Adverse effects did not occur. They concluded that treatment with NR was well tolerated and associated with improvement in ataxia and serum immunoglobulin concentrations in patients with A‐T.

### 6.3 Variable Selection in Untargeted Metabolomics and the Danger of Sparsity (Tinnevelt et al. 2020)

In this study, they included 14 patients with A‐T and treated them with 25 mg/kg NR. Metabolomics was used to measure as many metabolites as possible to identify biomarkers that could indicate disease mechanisms. Variable selection in chemometric methods was categorized into two groups: (1) sparse methods that identify the minimal set of variables needed to distinguish between groups, and (2) methods that identify all important variables for discrimination. These variables were summarized into metabolic pathways using pathway analysis tools. The metabolic effects of treatment with NR were studied in a cohort of patients with A‐T. Vitamin B3 serves as a significant cofactor for many enzymatic reactions in the human body. Therefore, it was anticipated that the variable selection method would identify vitamin B3 metabolites as well as other secondary metabolic changes during treatment. However, they did not select any vitamin B3 metabolites despite these metabolites showing a substantial difference when comparing intensity before and during treatment. Univariate analysis or significance multivariate correlation combined with pathway analysis was able to select vitamin B3 and other metabolites. NR‐related pathways and metabolites significantly increased following NR supplementation.

### 6.4 Long‐Term Nicotinamide Riboside Use Improves Coordination and Eye Movements in Ataxia Telangiectasia (Presterud et al. 2024)

This study aimed to investigate the safety and benefits of long‐term NR supplementation in individuals with A‐T. It was a single‐arm, open‐label clinical trial performed in individuals with A‐T, receiving 500 mg NR over a period of 2 years. Ten A‐T patients completed the study. Biomarkers and clinical examinations were used to assess safety parameters. Standardized and validated neuromotor tests were used to monitor changes in neurological symptoms. Using generalized mixed models, test results were compared to expected disease progression based on historical data.

Results: NAD^+^ concentrations increased rapidly in peripheral blood and stabilized at a higher level than baseline. NR supplementation was well tolerated by most participants. The total scores in the neuromotor test panels, as evaluated at the 18‐month time point, improved for all but one participant, primarily driven by improvements in coordination subscores and eye movements. A comparison with historical data revealed that the progression of certain neuromotor symptoms was slower than anticipated.

Conclusions: Long‐term use of NR appears to be safe and well tolerated, and it improves motor coordination and eye movements in patients with A‐T of all ages. No serious adverse events. Longest NR supplementation trial to date.

## 7 Who Should Take NAD Supplementation

The above studies are small and limited in scope. While the number of patients is small, the findings mentioned are significant. It is challenging to collect patients with very rare diseases, and the numbers will always be small. While current studies on NAD supplementation in rare diseases are limited, they offer encouraging early results. Recent clinical studies (Berven et al. 2023) suggest that doses of 3 g NR per day appear safe in normal humans. In the above studies on rare diseases, much lower doses were used, and increased doses may improve clinical outcomes in future studies. The results are limited but hopeful, and there are plans to increase the number of patients and increase the doses in future studies. There are also plans to perform combination therapies using other promising compounds in addition to NR or NMN, including the addition of exercise as a regimen. It is important to pursue the studies, and since the individuals will be taking the compounds over many years, it is very important to continually ensure that there are no serious side effects.

Studies in rare diseases have been very valuable for understanding mechanisms and developing interventions. For many of these conditions there are no available treatments and helping these severely affected individuals is a major advance and the first priority, However, since the disease mechanisms in these disorders are central to the aging process and since the features in the conditions in many ways mimic normal aging, the benefits detected in these individuals may also be there in the normal aging process and therefore benefit the wider population.

There have been multiple human studies with NAD supplementation and in some cases, they have shown significant benefits and in others there was no difference between treatment and controls. Areas that seem particularly promising for this intervention are neurodegeneration, inflammation, synaptic transmission, vision, and hearing, whereas it has not been so effective in muscle function (Zhang, Wang, et al. 2025). An important goal of future work should be to identify the clinical areas and the particular individuals who would benefit the most from NAD supplementation. This area has to move toward precision medicine.

One of the challenges is to be able to determine the NAD levels in individuals and to identify those with lower baseline NAD. Another is to better understand the underlying mechanisms that deplete NAD levels in the cells. Many enzymes compete for the cellular pools of NAD (Strømland et al. 2021). These include sirtuins that are NAD+‐dependent deacetylases regulating gene expression and cellular stress responses. It also includes Poly (ADP‐ribose) polymerases (PARPs) that are involved in DNA repair; the PARPs consume NAD+ to modify proteins. It also includes CD38, a membrane‐bound enzyme that depletes NAD+ while regulating calcium signaling and NAMPTs, rate‐limiting enzymes in the NAD salvage pathway, essential for maintaining NAD+ levels, and NAD kinase that converts NAD+ into NADP+, and used in anabolic reactions (Campagna and Vignini 2023).

Given the excessive heterogeneity between NAD levels in tissues and cells, it is challenging to define how to assess the level of NAD in individuals relative to considering intervention. New methods are emerging for measuring NAD levels in blood and other tissues, including the brain. What would be desirable is to evaluate individuals for not only NAD levels but also the state of their NAD metabolism, but this is costly and complex. Some efforts have been made in assessing the NAD metabolome in clinical studies, and so far, variable effects are seen that do not in general correlate with the changes seen in NAD levels (Vinten et al. 2025).

A study analyzing plasma from healthy individuals found that while total NAD concentrations were similar between men and women, women had a higher NAD^+^/NADH redox ratio than men. This means that although both sexes had comparable amounts of NAD overall, the balance between its oxidized (NAD^+^) and reduced (NADH) forms leaned more toward NAD^+^ in women (Schwarzmann et al. 2021). Interestingly, this sex‐related difference in the redox ratio diminishes with age, especially when biological age markers like skin autofluorescence and pulse wave velocity are considered. So, while the total pool of NAD might not differ much, the way it is distributed between its active forms—and potentially how it's used in cellular processes—can vary between males and females (Schwarzmann et al. 2021).

## Author Contributions

V.B. conceived, wrote and edited the paper.

## Funding

The author has nothing to report.

## Conflicts of Interest

V.B. is an advisor for Niagen Biosciences.

## Data Availability

Data sharing not applicable to this article as no datasets were generated or analyzed during the current study.
